# Bias and sensitivity in numerosity perception of negative emotions among individuals with high social anxiety

**DOI:** 10.1038/s41598-022-15601-z

**Published:** 2022-07-04

**Authors:** Jae-Won Yang, Jongsoo Baek

**Affiliations:** 1 Depratment of Psychology, The Catholic University of Korea, Bucheon, Gyeonggi-do Republic of Korea; 2 Institute of Human Complexity and Systems Science, Yonsei University, 85, Songdogwahak-ro, Yeonsu-gu, Seoul, Incheon, 21983 Republic of Korea

**Keywords:** Human behaviour, Anxiety

## Abstract

The cognitive model of social anxiety suggests an association between social anxiety and cognitive bias toward negative social information. This study investigated the numerosity perception of emotional faces among individuals with high social anxiety. Seventy-five college students completed self-reported questionnaires—assessing social anxiety symptoms—and a numerosity comparison experiment. In each trial of the experiment, participants were presented with a group of 16 emotional faces, varying in the number of faces expressing positive and negative emotions. They were asked to judge which emotion—positive or negative—was more numerous in the crowd. Bias and sensitivity in numerosity perception of emotions were estimated by fitting a psychometric function to participants’ responses. Individuals with low social anxiety showed a bias toward positive faces (*t*(17) = 2.44, *p* = 0.026), while those with high social anxiety did not (*t*(17) = 1.87, *p* = 0.079). Correlation analyses indicated that social anxiety was negatively associated with the parameters of the function (mean for bias and standard deviation for sensitivity; *r* =  − 0.34*, p* = 0.003 for mean; *r* =  − 0.23*, p* = 0.047 for standard deviation). Thus, our results suggest that socially anxious individuals lack the bias toward positive emotion and are more sensitive to negative emotion than nonanxious individuals in perceiving the numerosity of facial expressions.

## Introduction

Social anxiety is the fear of being negatively evaluated by others in social settings^1^. The cognitive model of social anxiety suggests that social anxiety develops and is maintained by negative biases in social information processing^2,3^: individuals with high social anxiety have a bias—in selective attention, memory, and interpretation—toward negative stimuli or events.

Interpretational bias among people with social anxiety has been studied by examining how participants interpret a scenario about an ambiguous social situation^4,5^. In these studies, individuals with social anxiety tended to interpret social situations more negatively than those in the control group. Interpretational bias has also been studied using pictures of emotional faces. Since evaluations from others are often conveyed through and inferred from facial expressions, how individuals with social anxiety respond to others’ faces could provide insight into social information processing among these individuals. In previous studies wherein participants were presented with a picture of an emotional face and asked to identify its emotional category or rate its emotional magnitude, people with high social anxiety perceived neutral or ambiguous facial expressions more negatively than those in the control group^6–10^.

People often interact with more than one person on an everyday basis, and individuals experiencing social anxiety frequently report feeling anxious while interacting with a large group of people (e.g., presenting in front of a large audience)^11^. Furthermore, individuals with high social anxiety tend to evaluate the overall emotion of faces in the crowd more negatively than that of a single face^12^. Thus, studying the emotional perception of a group of faces would allow us to understand important characteristics of social information processing in social anxiety. Despite the significance of processing ensemble information, only a few studies have investigated the perception of a crowd of facial expressions among individuals with high social anxiety. In these studies, participants were asked to identify the emotional category that the average face belonged to or rate the average emotional magnitude^13,14^. Participants with social anxiety rated the average face at a higher negative emotional magnitude than did control participants.

Another essential aspect of visual information processing of a group of stimuli is the numerosity perception^15^, which refers to the ability to estimate the quantity of objects. As mean and numerosity are mathematically related to each other (i.e., the mean is the sum of all element values divided by the numerosity), it is essential to measure the numerosity perception of emotional faces for a better understanding of social information processing among individuals with high social anxiety. In a study investigating perceptual bias in the average emotion of a crowd of facial expressions, normal participants underweighted negative emotions and overweighed positive emotions, whereas participants with high social anxiety exhibited no such bias^16^. Although the authors did not measure the perceived numerosity directly, it could be considered equivalent to the term “weight”. Thus, the cognitive bias among people with high social anxiety could occur in the numerosity perception of emotional faces by either overestimating the number of negative faces or underestimating the number of positive faces. However, little is known about the characteristics of numerosity perception of emotional information among people with high social anxiety.

**Figure 1:**
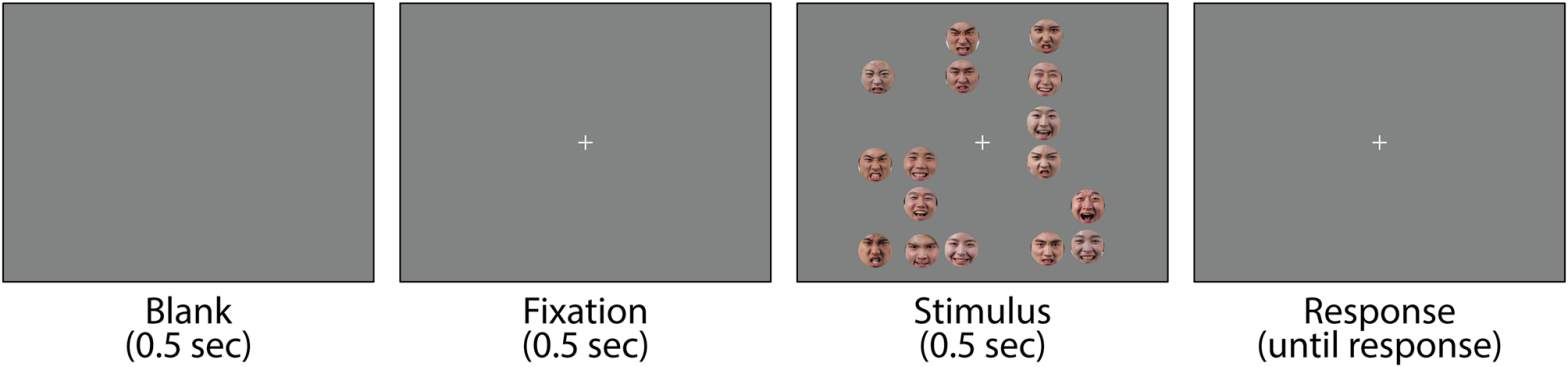
Illustration of the experimental procedure. Following a central fixation, a crowd with 16 emotional faces was presented to the participants. Participants’ task was to determine which positive or negative emotional faces were more numerous in the stimulus display.

The goal of this study was to characterize how individuals with high social anxiety perceive the emotional expressions of a crowd of faces, especially concerning numerosity perception. The characteristics of numerosity perception can be described using two indices: bias and sensitivity. Bias indicates the difference between the presented and perceived numbers of emotional faces. It is often measured using the actual number of emotional faces at which a participant subjectively perceived crowds as portraying a balance of emotions (i.e., perceiving half of the faces in the crowd as positive and the other half as negative). Sensitivity refers to the degree of precision in emotional judgments. It indicates how rapidly the perceived number of negative faces increases with an increase in the actual number. We aimed to determine the measure(s) on which individuals with high social anxiety might differ from their counterparts. To estimate the perceived number of emotional faces, we conducted a numerosity comparison task. Participants were presented with a group of pictures that included 16 emotional faces (Fig. 1). The number of faces expressing positive (happy) and negative (angry) emotions varied with each trial. Participants were instructed to report which emotion (positive or negative) was more numerous in the given pictures. We examined if the emotional numerosity perception of clinical samples with the social anxiety disorder, or their equivalent, differed from that of the normal population. Participants were grouped into two social anxiety groups: those with social anxiety levels above the clinical cutoff as the high social anxiety group (HSA) and their counterparts as the low social anxiety group (LSA). Then, we compared the proportion of negative responses (i.e., reporting that negative faces were more numerous than the positive faces) and the summary indices of numerosity perception (i.e., bias and sensitivity) between both groups. We also investigated the relationship between social anxiety and characteristics of numerosity perception with a correlation analysis for all participants. It has been claimed that social anxiety is a common symptom for normal and special populations and that the social anxiety of both populations is different quantitatively, not qualitatively^17^. Therefore, our results regarding the relationship between social anxiety and numerosity perception could be more generalized by analyzing all participants’ data rather than focusing on two extreme social anxiety groups.

## Results

Participants’ (*N* = 75) social anxiety levels were assessed using the Social Interaction Anxiety Scale^18^ (SIAS) and the Brief Fear of Negative Evaluation scale^19^ (B-FNE). The average SIAS score of the participants was 27.96 (*SD* = 14.07), and the average B-FNE score was 35.76 (*SD* = 9.93). In this study, Cronbach’s *α*s were 0.93 for the SIAS and 0.93 for the B-FNE. The correlation coefficient between the SIAS and B-FNE was *r* = 0.74 (*p* < 0.001).

Participants with SIAS scores in the top and bottom quartiles were grouped into HSA (*n* = 18, SIAS ≥ 39, SIAS *M* = 47.33, *SD* = 6.95) and LSA (*n* = 18, SIAS ≤ 16, SIAS *M* = 11.06, *SD* = 3.56) social anxiety groups, respectively. The SIAS score of 40 (in the Korean version of SIAS) was reported as the best cutoff score, which showed the best discriminability between clinically high social anxiety and normal control groups (sensitivity = 0.87 and specificity = 0.87)^20^. In our study, the SIAS score in HSA was ≥ 39. Thus, the social anxiety level in HSA was almost equivalent to that of clinical samples. The average ages were 22.39 (*SD* = 2.59) in HSA and 22.67 (*SD* = 2.89) in LSA. There were four men (out of 18) in HSA and five men in LSA. Both groups did not significantly differ in age (*t*(34) = 0.30, *p* = 0.763) and sex (*χ*^2^ (1, *n* = 36) = 0.15, *p* = 0.700).

**Figure 2:**
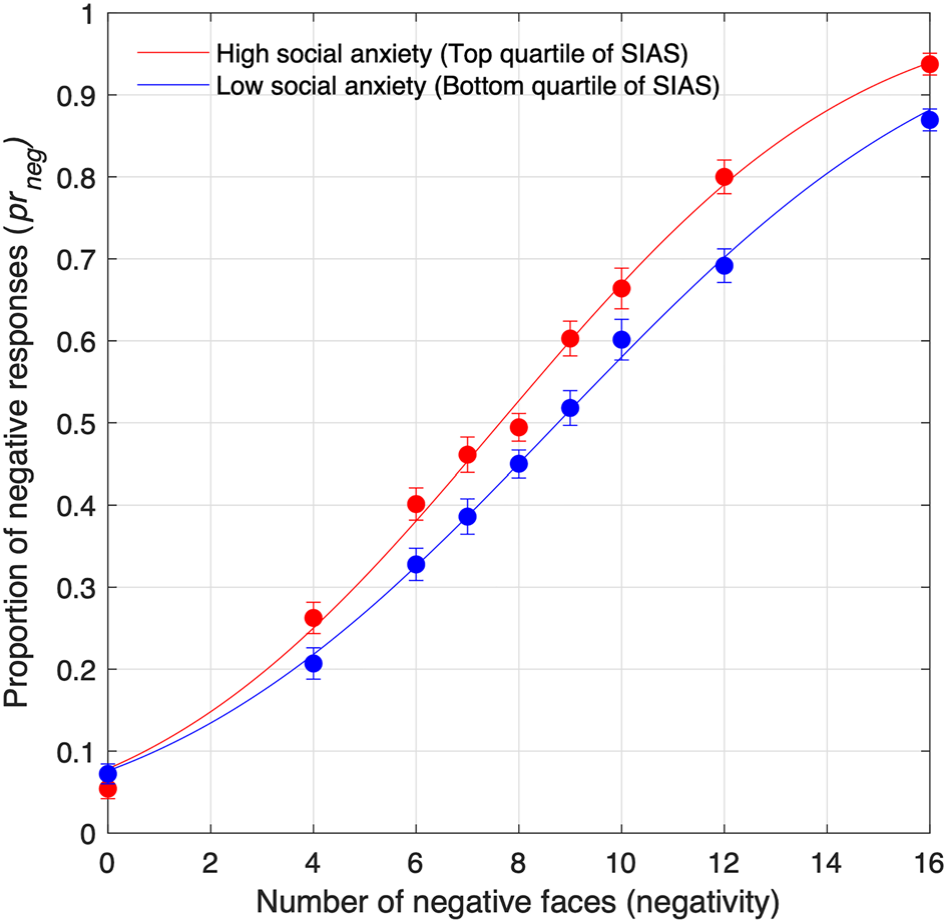
Proportion of negative responses (pr_neg_) in high and low social anxiety groups. The means (circles) were plotted as a function of the number of angry faces (negativity) along with fitted psychometric curves (solid lines). Error bars represent SEMs.

We examined the proportion of negative responses or *pr*_*neg*_ for each number of angry faces in the crowd (*negativity*). As shown in Fig. 2, *pr*_*neg*_ was equivalent in both groups when there were no negative faces (i.e., there were only happy faces) in the stimulus display. However, *pr*_*neg*_ of both groups differed in high negativity conditions. An analysis of variance (ANOVA) was conducted to test the effects of negativity and social anxiety on the proportion of negative responses. The main effects of negativity [*F*(8,272) = 426.83, *p* < 0.001, ${\eta }_{p}^{2}$ = 0.93] and social anxiety groups [*F*(1,34) = 10.00, *p* = 0.003, ${\eta }_{p}^{2}$= 0.23] were significant. The interaction between negativity and social anxiety groups was marginally significant [*F*(8,272) = 1.97, *p* = 0.051, ${\eta }_{p }^{2}$= 0.06]. Results of independent *t*-tests showed that differences in negative responses were significant for the negativity of 12 (Bonferroni corrected *p* = 0.045) and marginally significant for the negativity of 16 (Bonferroni corrected *p* = 0.063). There was no significant difference between groups in other negativity levels.

To characterize numerosity perception of emotional faces, we fitted a cumulative normal function to data—*pr*_*neg*_ as a function of negativity—at the individual level for each participant. The mean of the psychometric function (*μ)* indicates the actual number of angry faces where a participant perceives the same number of happy and angry faces in the display. If a participant’s *μ* is eight (i.e., the point of objective equality in which the actual numbers of faces expressing positive and negative emotions were equal), the participant’s numerosity perception is unbiased. A *μ* lower than eight indicates that the participant perceives angry faces more than happy faces for a display containing the same number of angry and happy faces, suggesting the presence of negative bias. A *μ* greater than eight, which indicates that a higher number of negative faces is perceived as the same as the number of positive faces, implies the presence of positive bias. The standard deviation of the psychometric function (*σ*) indicates the sensitivity of numerosity perception. When *σ* is small, the participant’s *pr*_*neg*_ increases rapidly as the number of angry faces increases. In contrast, *pr*_*neg*_ increases slowly when *σ* is large. In the model fitting, the average *μ* of the psychometric function was 8.16 (*SD* = 1.22), and the average σ was 5.60 (*SD* = 1.21). The overall goodness-of-fit was excellent (average *R*^2^ = 0.94, *SD* = 0.03).

The best-fitting models of both groups are presented using solid curves in Fig. 2. The average *μ* was 7.67 (*SD* = 0.76) in HSA, and 8.89 (*SD* = 1.55) in LSA. The average *σ* was 5.47 (*SD* = 1.18) in HSA, and 6.21 (*SD* = 1.77) in LSA. Result of *t*-tests showed that there was a significant difference in *μ* (*t*(34) = 3.01, *p* = 0.005) but not in *σ* (*t*(34) = 1.49, *p* = 0.146). Interestingly, *μ* was significantly higher than the point of objective equality (i.e., negativity = 8) in LSA (*t*(17) = 2.44, *p* = 0.026). These indicated that the number perception of LSA was biased positively. In contrast, *μ* was not significantly different from the point of objective equality in HSA (*t*(17) = 1.87, *p* = 0.079). The positive bias observed in LSA was not found in HSA.

**Figure 3:**
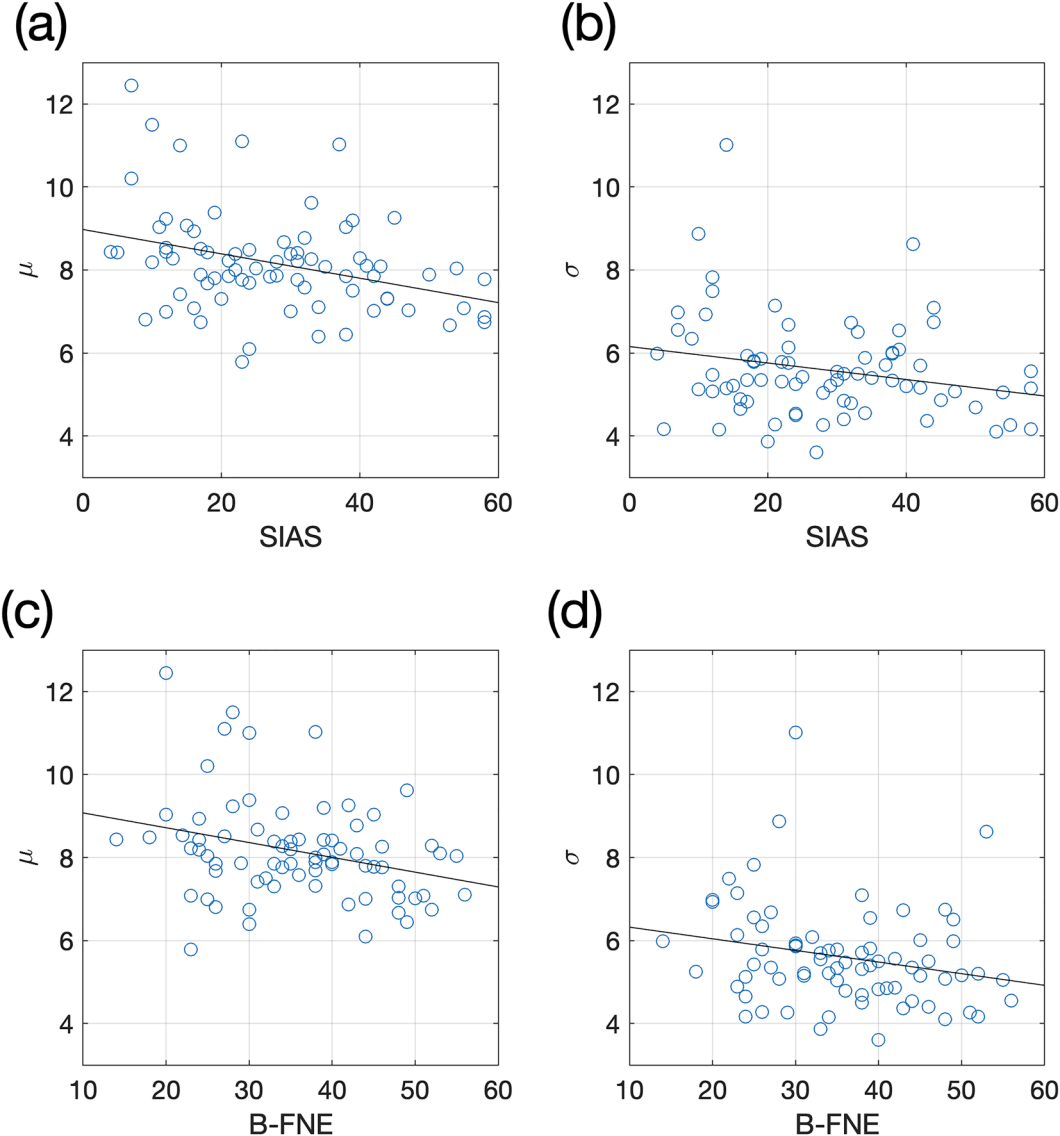
Social anxiety scores versus parameter estimates. Each data point represents one participant and solid line represents regression line in each panel. Both (**a**) SIAS and (**b**) B-FNE scores were negatively correlated with the mean of the psychometric function (μ). Both (**c**) SIAS and (**d**) B-FNE scores were also negatively correlated with the slope of the psychometric function (σ).

Then, a correlation analysis was conducted to examine the relationship between the levels of social anxiety (SIAS and B-FNE scores) and the characteristics of numerosity perception of emotional faces (parameters of the psychometric function: *μ* for bias and *σ* for sensitivity) for all participants. The bias in numerosity perception for emotional faces (*μ*) was significantly associated with social anxiety indices (*r* =  − 0.34*, p* = 0.003 for SIAS; *r* =  − 0.29*, p* = 0.011 for B-FNE). High levels of social anxiety were associated with a tendency to perceive more negative faces with lower *μ* (Fig. 3a for SIAS and 3c for B-FNE). The sensitivity of numerosity perception of facial expression (*σ*) was also significantly associated with the social anxiety indices (*r* =  − 0.23*, p* = 0.047 for SIAS; *r* =  − 0.23*, p* = 0.048 for B-FNE). Participants with high social anxiety showed greater sensitivity in numerosity perception (Fig. 3b for SIAS and 3d for B-FNE). In sum, the levels of social anxiety were negatively associated with two parameters of the psychometric function: bias and sensitivity.

## Discussion

This study explored the numerosity perception of emotional faces in individuals with high social anxiety. In the numerosity comparison task with varying numbers of positive and negative faces, participants were asked to decide which emotional category was more numerous in the stimulus display. The proportion of negative responses rose with the increasing number of negative faces. Although the proportion of negative responses in HSA and LSA did not differ in trials containing only positive faces (negativity = 0), the difference became greater when negativity was high. The proportions were significantly higher in HSA than in LSA for high negativity levels. Overall, the numerosity perception was biased toward positive stimuli in LSA but not in HSA. Individuals with a higher level of social anxiety perceived the crowds more negatively than their less anxious counterparts because the nonanxious individuals showed a positive bias, whereas socially anxious individuals lacked the positive bias. This finding is in accordance with studies reporting the “lack of positive bias” in social information processing among socially anxious individuals^14,21^. However, in another study^13^, both groups showed negative bias: more negatively in HSA and less negatively in LSA. Nevertheless, findings from all these studies consistently indicated that bias was more negative in HSA than in LSA.

To examine the characteristics of numerosity perception in social anxiety, we fitted the proportion of negative responses vs. negativity to a cumulative normal function to find the best-fitting parameters of the psychometric function (*μ* for bias and *σ* for sensitivity). Then, a correlation analysis was conducted between the parameters and the level of social anxiety (indicated by the SIAS and the B-FNE scores). Both bias and sensitivity of the function were negatively correlated with the social anxiety indices. Our findings implied that people with high social anxiety tended to perceive more negative faces as compared to people with low social anxiety, and the perceived number of negative faces increased more sensitively with an increase in the number of negative faces.

The two analyses outlined in the current paper showed different results about the relationship between sensitivity and social anxiety: they were significantly associated with each other in the correlation analyses, while there was no statistical difference in sensitivity between HSA and LSA in the group comparison with *t*-tests. A possible explanation for this discrepancy is the loss of information and statistical power caused by splitting a continuous variable (SIAS scores) into a categorical variable (anxiety groups) in the group comparison.

Why do people with high social anxiety tend to perceive the number of emotional expressions differently from their counterparts? People with high social anxiety negatively evaluate the emotional value of facial expressions even when these expressions are presented individually^6–10^. Thus, the negative bias for an individual face could result in the overestimation of the number of negative faces among a group of emotional expressions. However, considering that the current study used full-blown expressions as stimuli and that a bias in perceiving a single face was observed for ambiguous or neutral faces rather than full-blown expressions^7,22^, it is less likely that participants with high social anxiety perceived an extremely happy face as an angry face. Hence, our findings suggested that the bias in the perceived emotional value of an individual face was not the main reason for the bias in the perceived number of negative faces. Another possibility is that participants responded based on the average perception rather than the numerosity perception in this experiment. Although they were explicitly asked to compare the numbers of two emotional faces, they could use the strategy of responding the negative faces were more numerous than the positive ones when the perceived average emotion was negative and vice versa. Unfortunately, the current experimental design could not distinguish participants’ internal strategies; however, there seemed to be distinct signature patterns between the average and numerosity perceptions of emotional faces. In our previous study^14^, we investigated the average emotional strength of a facial crowd using a similar experimental design and stimuli to the current study. The results showed that social anxiety was negatively associated with bias but not with sensitivity in perceiving the average emotional strength of the facial crowd. Furthermore, the difference in negative responses between participants with high and low social anxiety was significant even under lower negativity conditions. Thus, if participants responded based on their perceived average emotion in the current study, we would expect a difference in negative responses between both groups under lower negativity conditions and no association between social anxiety and sensitivity of numerosity perception. However, the current results showed that social anxiety was also negatively associated with sensitivity, and the proportion of negative responses was almost the same in both groups under lower negativity conditions. Considering the different data patterns between these studies, we could exclude the possibility of the average-based responses in this experiment and conclude our finding is a separate phenomenon from the average perception of facial crowds among individuals with social anxiety.

The negative bias in numerosity perception among people with social anxiety could have resulted from bias in selective attention. In an eye-tracking study, people with high social anxiety fixated their eye positions more frequently on negative faces than on positive ones, although the difference was non-significant^23^. In another study, individuals with high social anxiety responded faster to stimuli presented at the location of the negative face than to those at the location of the positive face^24^. These findings implied that they focused more frequently on—and processed more in the perceptual stage—socially negative stimuli than positive stimuli. Thus, attentional bias could account for the bias in numerosity perception. Biased selection can also occur in working memory. Considering the limited capacity of visual working memory, which is only a few items^25^, people might not be able to take all or many social inputs into account. Instead, they might selectively consider only a limited number of samples to form an ensemble perception, and the remaining samples fleet away. Thus, the negatively biased samples retained in working memory could be a possible cause of bias in the numerosity perception of emotional faces in the crowd. Since specific mechanisms underlying the bias and precision in numerosity perception of emotional faces among individuals with social anxiety are still unknown, further research is required.

What is the relationship between the numerosity and the average perception of a crowd of facial expressions? People with high social anxiety tend to evaluate the average emotional value of a facial crowd more negatively than that of an individual face^12^. The more negative evaluation of the average emotion of the facial crowd could be caused by bias in the numerosity perception. As compared to the normal control group, people with high social anxiety tend to miss positive faces more often, omitting them more frequently from the later information integration stages (such as summation or averaging), resulting in a more negative bias in the estimated mean than in perceiving a single face. However, the perception of an individual face, the numerosity perception, and the average emotional perception of a facial crowd could be complexly entangled. To fully understand the characteristics of information processing in social anxiety, a comprehensive model overarching all these element terms and an experimental framework validating the model should be developed in future studies.

## Methods

Participants completed questionnaires assessing their level of social anxiety via a web-based survey. They then participated in a psychophysical experiment in which numerosity perception of emotional faces was measured through an online experiment site. It took approximately 20 min to complete both the questionnaires and the experiment. Written informed consent was obtained from all participants at the beginning of the survey. The study procedure was reviewed and approved by the Institutional Review Board of the Catholic University of Korea. The work was conducted in accordance with the Declaration of Helsinki.

### Participants

The sample size was determined by a power analysis. In our previous studies, in which we explored the relationship between the levels of social anxiety and emotional perception of facial crowds, the correlation coefficients were 0.25^14^, 0.35^26^, and 0.38^27^. Based on an effect size of 0.35 (median of the correlation coefficients in these studies) with a 0.95 power and *α* = 0.05, a sample size of 83 was required to identify the relationship between the two variables. Thus, we recruited 83 participants for the experiment but removed two participants’ data because one participated in the experiment using a mobile phone, and the other did not complete the questionnaire.

Eighty-one South Korean undergraduate students participated in the study in exchange for monetary compensation of 2000 KRW (approximately $2.00 USD). It has often been noted that low reliability is a limitation of online studies. To avoid this issue, we excluded outliers using two criteria. First, we explored each participant’s data and the fitting results. One participant gave seemingly random responses, such that *pr*_*neg*_ was close to the chance level (0.5) across all conditions, and the goodness-of-fit was − 0.01. Thus, we excluded this participant from the data analysis. The second criterion was the error rate in catch trials. In this study, the catch trials were defined as trials with only angry (negativity = 16) or happy (negativity = 0) faces. The error rate in catch trials (i.e., the proportion of negative responses for the display containing only happy faces and positive responses for display containing only angry faces) was very low for most participants (M = 0.08, SD = 0.08). However, five participants showed an error rate greater than 0.23, which corresponds to a mean of + 2SD. We excluded these participants from further analyses. Therefore, the following analyses were based on the data of the remaining 75 individuals (57 women and 18 men; M_age_ = 22.59 years, SD = 2.63 years). The final mean error rate for catch trials was 0.06 (SD = 0.06).

### Measures of social anxiety

Participants’ social anxiety levels were assessed using the SIAS^18^ and the B-FNE^19^. The SIAS included 20 statements describing distress experienced during interaction with others. The B-FNE scale consisted of 12 statements evaluating the fear of negative evaluation by others. Thus, the SIAS assesses the level of social anxiety, while the B-FNE measures an underlying cognitive component of social anxiety. For both scales, participants were instructed to indicate the degree to which each statement described themselves on a 5-point Likert scale (ranging from 0 to 4 in the SIAS and from 1 to 5 in the B-FNE scale). In this study, we used the Korean versions of the SIAS^28^ and the B-FNE scale^29^. A high score on both scales indicated a high level of social anxiety.

### Psychophysical experiment for estimating numerosity perception of facial expression

We conducted a psychophysical experiment with a numerosity comparison task to characterize participants’ numerosity perception of emotional faces. The program was coded using PsychoPy v2021.1.4^30^ and hosted on an online repository for psychological experiments. Participants ran the experiment by accessing the site through a web browser.

A group of full-blown emotional faces—either angry or happy—was presented on gray background in each trial. Images were obtained from the Yonsei Face Database (YFace DB)^31^. In the development of YFace DB, all pictures were validated for accuracy (correct categorization of each facial picture to seven basic emotional categories), intensity, and naturalness by 221 participants. Results showed excellent accuracy (99.02% correct for happy and 86.71% for angry) and strong intensities (5.34 for happy and 5.37 for angry on a 7-point Likert scale). Thus, stimuli in our experiment were representative faces for each emotional category. We obtained permission from the copyright holder to use and publish the facial images. Out of 24 models (12 women and 12 men) in the database, 16 were randomly selected for each trial. Emotional faces (128 × 128 pixels each) were randomly presented on an invisible 6 × 6 grid (960 × 960 pixels), excluding the central 2 × 2 grid. In each cell of the grid, the position of each picture was randomly jittered—within a range of 10 pixels along the horizontal and vertical axes—independently for each trial.

The number of angry faces in the stimulus display varied across nine levels (with 0, 4, 6, 7, 8, 9, 10, 12, and 16 angry faces) with the remainder being happy faces. All negativity conditions were randomly interleaved throughout the study for each participant. Trials were repeated 40 times for each condition resulting in 360 trials in total. A break was given after every 90 trials.

The experimental procedure is illustrated in Fig. 1. Each trial started with a fixation point of 0.5 s, followed by an image containing a group of faces, which was presented for 0.5 s. The task was to determine which emotional category of faces—positive or negative—was more numerous in the displayed image. Some participants were instructed to press the left arrow key on the keyboard when they thought there were more happy faces than angry faces and the right arrow key when they thought otherwise. For the other participants, the response keys were reversed; they were required to press the right arrow key if they thought that there were more happy faces and the left arrow key if they thought otherwise. Response key mapping was randomly assigned among participants. No feedback was provided.

### Data analysis

The group difference in demographic variables was analyzed using an independent *t*-test for age and chi-square analysis for sex. A mixed-model repeated-measures ANOVA was conducted to examine group differences in *pr*_*neg*_ for all negativity conditions with the negativity as a within-subject variable and the social anxiety group as a between-subject variable. To explore the simple main effects of the social anxiety group, we compared *pr*_*neg*_ of both groups at each negativity condition separately using independent samples *t*-tests with a Bonferroni correction. Then, the cumulative normal distribution was fitted to the measured *pr*_*neg*_ as a function of negativity. The best-fitting parameters (*μ* for bias and *σ* for sensitivity) were searched for each participant using a least-square procedure, which minimized the least-squares difference between the measured and the predicted *pr*_*neg*_. The fitting procedure was implemented with MATLAB Curve Fitting toolbox (Mathworks, Natick, MA, USA). The difference in the best fitting parameters between both groups was tested with independent *t*-tests. Furthermore, we conducted a one-sample *t*-test to compare *μ* against 8 for each group to test whether the numerosity perception was biased. No priori power calculation was performed for the group comparisons with *t*-tests and ANOVA. In addition, four sets of correlation analyses were performed to study the relationship between the social anxiety level (SIAS and B-FNE) and the characteristics of numerosity perception (*μ* and *σ*). In these analyses, all 75 participants’ data were included. To identify outliers, we inspected Cook’s distance^32^, an estimate of the influence of each data point, for all participants. No outlier was detected with a cut-off value of 1 for Cook’s distance.

### Ethics declarations

The study procedures were reviewed and approved by the Institutional Review Board of the Catholic University of Korea.

## Data Availability

All datasets, questionnaires, and codes for the experiment are available to download at a public repository (https://osf.io/zq3wf).
